# *Alysicarpus poklianus* (Fabaceae, Desmodieae), a new species from India

**DOI:** 10.3897/phytokeys.68.9975

**Published:** 2016-08-16

**Authors:** Akram Gholami, Arun K. Pandey

**Affiliations:** 1 Department of Botany, University of Delhi, Delhi-110007, India

**Keywords:** Alysicarpus, endemic new taxon, taxonomy, India

## Abstract

A new species, *Alysicarpus poklianus* Gholami & Pandey from Sinhgarh, Maharashtra, India is described. It is morphologically most similar to *Alysicarpus hamosus* but differs in having ovate leaves, rounded-ovate bracts, larger size of calyx, pods comprising 5–7 longer than broad joints with easily separable septa. In this study, a comprehensive description, and identification key of *Alysicarpus poklianus* are provided.

## Introduction

The genus *Alysicarpus* Necker ex Desvaux, a member of tribe Desmodieae, family Fabaceae, comprises approximately 30 species distributed in tropical and subtropical regions of the old world (Lewis et al. 2005).The major centers of diversity of the genus are Africa (10 spp.), India, Indo-China, Malaysia and Japan (20 spp.) (Lewis et al. 2005, Mabberley 2009). In India, the genus is represented by approximately 18 species, (Sanjappa 1992, Pokle 2002, Gholami and Pandey 2016).

The genus *Alysicarpus* is characterized by its calyx with reticulate or striate venation, and turgid indehiscent pods. The leaves are generally unifoliolate or rarely pinnately 3-foliolate (Pedley 2001, Pokle 2002).

During the taxonomic revision of the genus *Alysicarpus*, field trips were made to different parts of India and several specimens were collected. We compared our collected specimens with all the voucher specimens of *Alysicarpus* species deposited in different herbaria (BAMU, BSD, BSI, CAL, DD, DUH, LWG, MH, PAN). A critical examination of the collected specimens and literature indicated that the collected material represented an undescribed species. Hence, the objective of the present study was to undertake morphological and molecular analyses to test whether these specimens represent a new taxon. Our unpublished preliminary molecular sequence data analysis supports the recognition of new species. In the present communication, the new species is described based on morphological data supplemented with identification key for all Indian species of *Alysicarpus*.

## Materials and methods

### Morphology

The overall morphology of the new species was examined by stereobinocular microscope (SMZ 1000). For morphological comparisons, we consulted herbarium specimens kept in different herbaria in India (BAMU, BSD, BSI, CAL, DD, DUH, LWG, MH, PAN). The Flora of India and floras of different states in India and neighboring regions including China, Bhutan, Nepal, Pakistan, Bangladesh were also consulted. The diagnostic traits of the new species and morphologically most similar species viz., *Alysicarpus hamosus* and *Alysicarpus ovalifolius* are presented in Table 1. In addition, an identification key is provided to distinguish new species and other taxa.

**Table 1.** Differences between *Alysicarpus poklianus*, *Alysicarpus hamosus* and *Alysicarpus ovalifolius*.

Characters	*Alysicarpus poklianus* sp. nov.	*Alysicarpus hamosus*	*Alysicarpus ovalifolius*
Stem	Densely covered with long hairs	Densely covered with long hairs	Glabrous or sparsely covered with short hairs
Leaves	Unifoliolate	Unifoliolate mixed with trifoliolate	Unifoliolate
Leaflet shape	Ovate to orbicular	Orbicular	Ovate at base, lanceolate in the upper part
Leaflet size	10–50 × 5–40 mm	5–20 × 5–20 mm	20–60 × 10–20 mm
Inflorescence	50–150 mm long	30–40 mm long	50–150 mm long
Pedicel	2–5 mm long, filiform	1–3 mm long, thick	1–2 mm long, thick
Calyx	3–5 mm long	1–3 mm long	4–6 mm long
Pod size	15–20 × 2–3 mm	10–15 × 2–3 mm	15–20 × 2–3 mm
Pod joints	5–7 joints, longer than broad	3–5 joints, broader than long	5–8 joints, longer than broad
Pod septa	Present, easily separable	Present, not easily separable	Septa absent
Spermoderm	Rugulate	Rugulate	Foveo-rugulate

### SEM study

For SEM study, mature seeds were mounted on aluminum stubs with double adhesive tape and sputter-coated with gold palladium in a JFC-1600 Autofine coater, JEOL, Japan sputter coating unit. Samples were examined using a Scanning Electron Microscope JSM-6610LV, JEOL, Japan, at the Department of Botany, University of Delhi, India.

## Results

### Morphology

*Alysicarpus poklianus* is distinct from *Alysicarpus hamosus* in having ovate leaves, longer pods with easily separable septa and foveo-regulate pattern of spermoderm. Table 1 gives an overview of the differences between *Alysicarpus poklianus* (the new species proposed here), *Alysicarpus hamosus* and *Alysicarpus ovalifolius*.

### Taxonomic treatment

#### Alysicarpus poklianus

Taxon classificationPlantaeFabalesFabaceae

A. Gholami & A. K. Pandey sp. nov.

urn:lsid:ipni.org:names:60472842-2

Fig. 1 , 2

##### Note.

Diagnostic characters for *Alysicarpus poklianus* include ovate leaves, 5–7 joint pods, easily separable septa and foveo-regulate spermoderm.

##### Type.

INDIA. Maharashtra: Sinhgarh, 18°21’56.39”N, 73°45’18.97”E, 587 m, 19 October 2014, *Gholami & Pandey 4642* (holotype DUH!, isotype BSD!, CAL!).

##### Description.

Annual, prostrate to procumbent, profusely branched, slender, 30–50 cm long, stem densely covered with long hairs.Stipules triangular to linear, scarious, acute striate, 3–7 mm long, glabrous with ciliate margins. Petiole 5–6 mm, hairy. Leaflets ovate to oval, 15–35 × 5–20 mm, apex rounded to mucronulate, both surfaces hairy though the density of hair on lower surface is more. Inflorescence axillary or terminal, 50–150 mm long, lax with stiff hairs. Flowers in pairs, 2–3 pairs along each rachis, subtended by deciduous bract and secondary bracts; pedicels 3–5 mm long. Bracts rounded to ovate, 3–4 mm long, acute, ciliate at margins with long hairs; secondary bracts 1–3 mm long, lanceolate to triangular, ciliate at margins with long hairs. Calyx much shorter than the first joint of the pod; 2–3 mm long, tube very short, c. 1 mm long, lobes acute, not imbricated, ciliate all over. Standard petal light pink, 2–3 mm long, emarginated at apex; wing petals dark pink, 3–3.5 mm long; keel petal boat-shaped, bent and folded, c. 3 mm long. Stamens diadelphous, 2–3 mm long. Ovary 1.5–3 mm long, pubescent. Pods cylindrical, 15–20 mm long, 1.5–2 mm broad, compressed, 5–7 jointed, clothed with straight and hooked hairs, septa between two joints of pod boat-shaped,easily separable. Seeds light to dark brown, 2 × 1 mm, oval, smooth, spermoderm rugulate.

**Figure 1.**
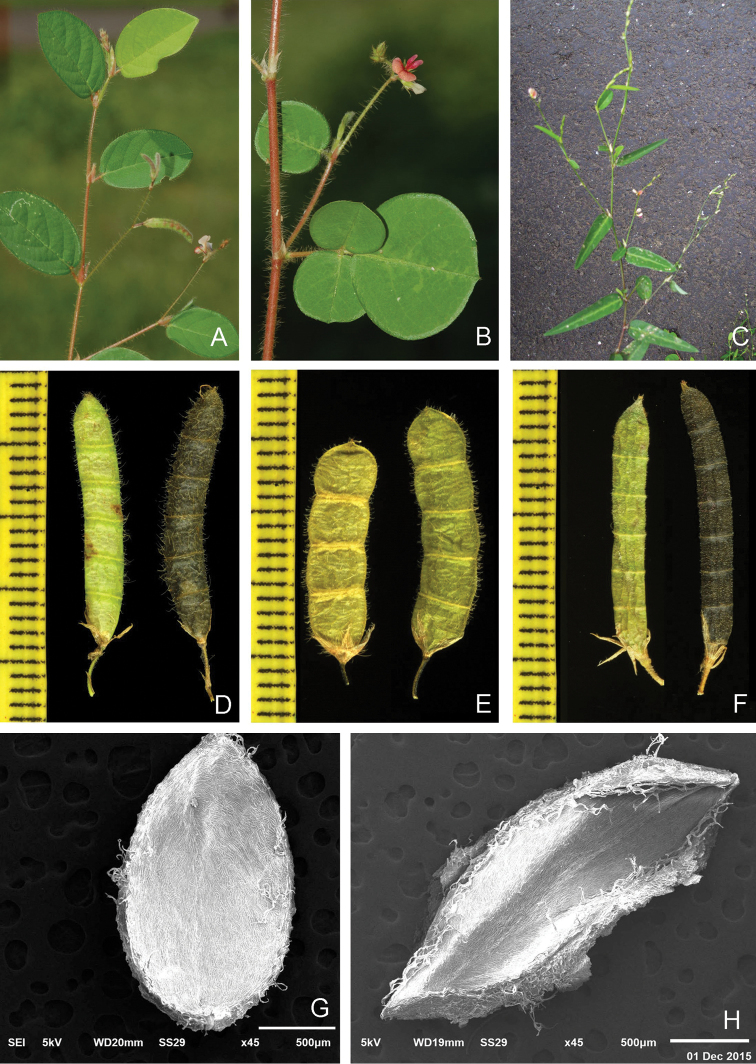
*Alysicarpus*. **A** *Alysicarpus poklianus* **B** *Alysicarpus hamosus* **C** *Alysicarpus ovalifolius* **D** Pod of *Alysicarpus poklianus* **E** pod of *Alysicarpus hamosus* **F** Pod of *Alysicarpus ovalifolius* **G, H** Pod septa of *Alysicarpus poklianus* and *Alysicarpus hamosus* respectively.

**Figure 2.**
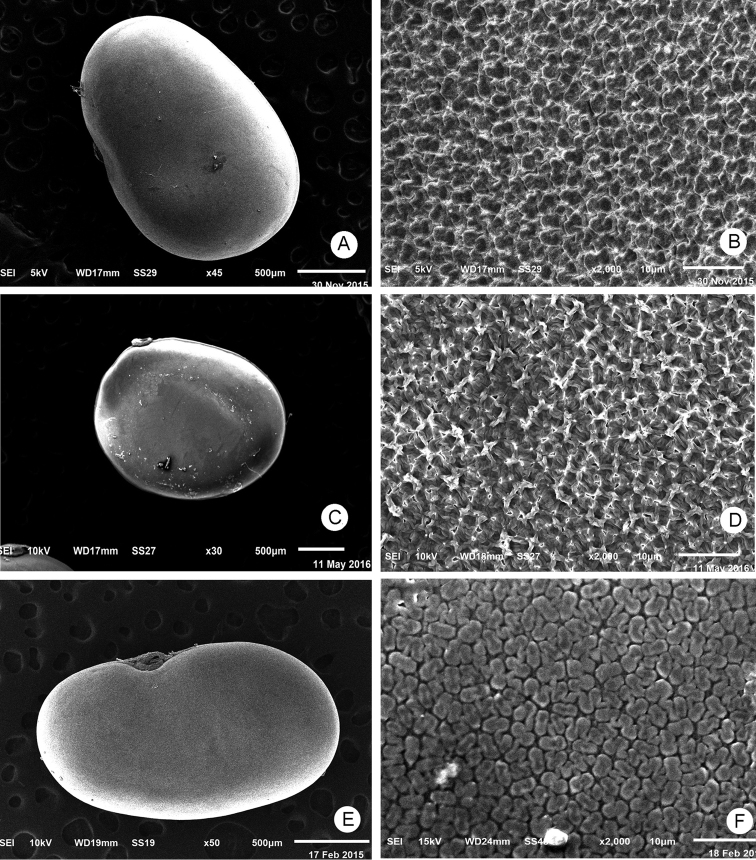
Seed and spermoderm pattern. **A, B** *Alysicarpus poklianus* **C, D** *Alysicarpus hamosus* **E, F** *Alysicarpus ovalifolius*.

##### Etymology.

The species is named in honor of Prof. D.S. Pokle who has done extensive work on the taxonomy of the genus *Alysicarpus* in India.

##### Distribution and habitat.

**Figure 3.**
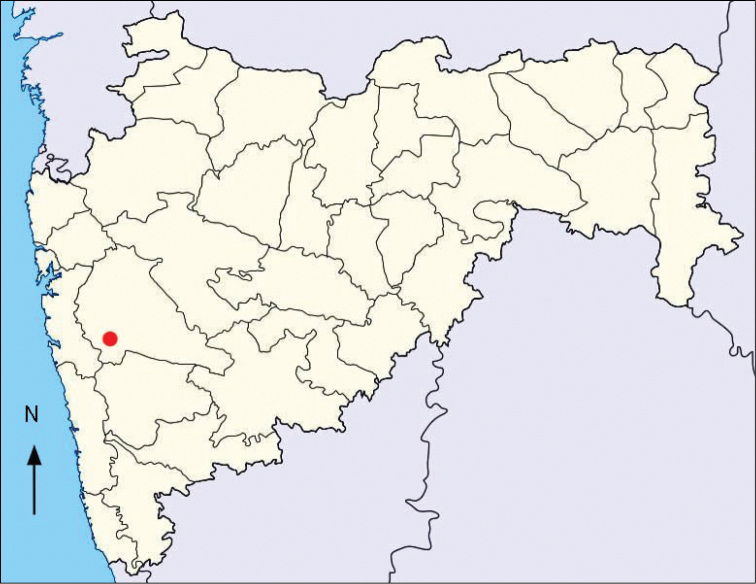
Distribution of *Alysicarpus poklianus* in Maharashtra, India

Maharashtra (Fig. 3), India, growing on gravely slopes along roadsides at 500–600 m elevation.

##### Phenology.

Flowering from August to October; fruiting from September to November.

#### Key for identification of *Alysicarpus* species in India

**Table:** 

1	Joints of pods strongly transversely rugose, never tetragonal.	**2**
–	Joints of pods tetragonal, rugose, reticulated or smooth	**5**
2	Inflorescence short dense, calyx and bract conspicuously ciliated, pod not exserted from calyx	***Alysicarpus scarious***
–	Inflorescence long dens or lax, calyx and bract less ciliated or glabrous, pod exserted from calyx	**3**
3	Secondary bract present	***Alysicarpus rugosus***
–	Secondary bract absent	**4**
4	Stem densely pubescent, calyx and bract slightly ciliate, leave ovate	***Alysicarpus heyneanus***
–	Stem glabrous or with a line of hair, calyx and bract glabrous, leaves usually linear-lanceolate	***Alysicarpus ludens***
5	Joints of pods reticulated or tetragonal rugose, calyx and bract densely covered with silky white hairs	**6**
–	Joints of pods slightly reticulate or smooth, hairs in calyx and bract not silky white	**8**
6	Leaflets 3-nerved at base, inflorescence long, pods included in the calyx	***Alysicarpus pubescens***
–	Leaflets 1-nerved at base, inflorescence short, podexserted from calyx	**7**
7	Joint of pod tetragonal, as long as broad, conspicuously reticulate	***Alysicarpus tetragonolobus***
–	Joint of pod 4-winged, longer than broad, obscurely reticulate	***Alysicarpus luteovexillatus***
8	Calyx reticulate-veined, shorter than first joint of pod	**9**
–	Calyx striate-veined, longer than first joint of pod	**13**
9	Pod moniliform	***Alysicarpus monilifer***
–	Pod cylindric not moniliform	**10**
10	Pod not pubescent or hairs are short	**11**
–	Pod conspicuously pubescent with long hair	**12**
11	Infructescence lax, leaflets dimorphic	***Alysicarpus ovalifolius***
–	Infructescence dense, leaflets uniform	***Alysicarpus vaginalis***
12	Pod 3–5 joint, joints of pod broader than long, pod septa not easily separable, leaflets orbicular, mix one and three foliolate	***Alysicarpus hamosus***
–	Pod 5–7 joint, joints of pod longer than broad, pod septa easily separable, leaflets ovate, one foliolate	***Alysicarpus poklianus***
13	Pod puberulous, calyx and bract densely pubescent, secondary bract absent	***Alysicarpus longifolius***
–	Pod glossy, glabrous, calyx and bract slightly ciliate at the margin, secondary bract present	
14	Pedicel 3–4 mm long filiform, pod drooping	***Alysicarpus gamblei***
–	Pedicel 1–2 mm long, pod straight	**15**
15	Pod moniliform, branches glabrous, leflets elliptic oblong	***Alysicarpus gautalensis***
–	Pod cylindrical, branches covered with appressed hairs	**16**
16	Leaflets linear lanceolate, calyx and bract almost glabrous	***Alysicarpus bupleurifolius***
–	Leaflets usually ovate-obovate, calyx and bract more ciliate	***Alysicarpus naikianus***

#### Additional specimens examined

INDIA, Maharashtra, Nanded, Bodhadi, 14.12.1997, *A. S. Dhabe*, *913* (BAMU); Aurangabad, 13.10.1998, *A. S. Dhabe, 948* (BAMU); Satara, 20.09.1998, *Ravi Patil*, 236 (BAMU); Poona, 2.8.1960, *John Cherian* 63517 (CAL).

## Supplementary Material

XML Treatment for Alysicarpus poklianus
